# Differential cardiac impacts of hematological malignancies and solid tumors: a histopathological and biomarker study

**DOI:** 10.1186/s40959-024-00285-3

**Published:** 2024-12-19

**Authors:** Michael H. Udin, Sunitha Shyam Sunder, Sarmila Nepali, Sharma Kattel, Amr Abdelradi, Scott T. Doyle, Ciprian N. Ionita, Qian Liu, Umesh C. Sharma, Saraswati Pokharel

**Affiliations:** 1 Department of Pathology and Laboratory Medicine, Roswell Park Comprehensive Cancer Center, Buffalo, NY USA; 2 Department of Medicine, Division of Cardiovascular Medicine, University of Minnesota, Minneapolis, MN USA; 3 Division of Cardiology, Department of Medicine, School of Medicine and Biomedical Sciences, University at Buffalo, Buffalo, NY USA; 4 Department of Biomedical Engineering, University at Buffalo, Buffalo, NY USA; 5 Canon Stroke and Vascular Research Center, Buffalo, NY USA; 6 Department of Biostatistics and Bioinformatics, Roswell Park Comprehensive Cancer Center, Buffalo, NY USA; 7 Department of Medicine, School of Medicine and Biomedical Sciences, University at Buffalo, Buffalo, NY USA

**Keywords:** Cardiotoxicity, Cancer, Cancer therapy, Hematological malignancy, Solid tumor, Heart failure, Fibrosis, Collagen, Second harmonic generation, Acute myeloid leukemia

## Abstract

**Background:**

Cancer patients are known to be associated with increased risk of cardiovascular disease. However, no studies have examined the differential impact of hematologic malignancies (HMs) and solid tumors (STs) on cardiac morphology at the tissue level.

**Objective:**

We aimed to examine histopathological features alongside cardiovascular biomarkers in patients with HMs and STs who underwent post-mortem evaluation.

**Methods:**

We analyzed cardiac changes in 198 patients with HMs and 164 patients with solid tumors STs. We compared demographics, echocardiogram data, exposure to various antineoplastic agents, and post-mortem findings. Additionally, cardiac histological validation was conducted on post-mortem cardiac specimens to examine cardiac tissue morphology, focusing on cardiomyocyte nuclear density, collagen content, and collagen fiber orientation.

**Results:**

HM patients displayed significantly disordered collagen fiber alignment (0.71 vs 0.83, *P* = 0.027), and reduced cardiomyocyte nuclear density (56 vs 72, *P* = 0.002) compared to ST patients. Similarly, hemoglobin level was decreased (6.71 vs 8.06, *P* < 0.001) in HM patients compared to ST patients. HM patients also showed elevated B-type natriuretic peptide levels (2,275 vs 867, *P* < 0.001), without significant differences in creatine-kinase MB and cardiac troponin levels. Multivariate analysis identified increased right ventricular thickness, low diastolic blood pressure, and high cardiac troponin levels as risk factors for cardiac death in HM patients.

**Conclusions:**

This study demonstrates that HM patients have fewer cardiomyocyte nuclei and poorly aligned collagen, with serum biomarker evidence of increased cardiac dysfunction. This supports the necessity for specialized cardiac care for these patients.

## Introduction

Adverse cardiovascular effects (ACEs) in cancer patients are a growing public health concern. With advancements in cancer therapy contributing to improved survival, particularly among hematological malignancy (HM) patients, and with populations increasing in size and age, the number of people facing ACEs has continued to expand [1, 2]. Due to this expansion, there is an increasing need to investigate the underlying causes of these ACEs. Particularly, there is a lack studies examining the differences in the impact of HMs and solid tumors (STs) on cardiovascular complications, especially as it relates to biomarkers of cardiac injury and myocardial tissue morphology.

HMs are systemic diseases and can impact areas directly connected to circulatory pathways in contrast to STs, which are generally localized masses. Voight et al. highlighted the systemic nature of HMs through finding a significant association between cardiac infiltrative disease and B-cell malignancies [3]. Due to the systemic way in which HMs impact the body vs STs, there is a need to conduct research aimed at better understanding their biological mechanisms and impact on the heart. In particular, previous studies have reported a high prevalence of cardiovascular dysfunction associated with HMs, with these malignancies showing the highest instance of heart failure deaths among all cancer subtypes [4, 5]. There are varying direct and indirect impacts of HMs on cardiovascular health. Some studies have shown that leukemias may lead to secondary cardiovascular strain and immunosuppression, while lymphomas may induce cardiotoxicity through systemic effects such as cardiac infiltration and anemia [6–9]. Additionally, there is evidence that acute myeloid leukemia (AML) triggers an inflammatory response that can adversely affect cardiac function [10]. These findings highlight the complex relationship between HMs and cardiovascular health and suggest a need for research that explores these differences to aid in development of clinical surveillance strategies and targeted interventions.

To address these challenges, we conducted a comparative analysis using data from Roswell Park Comprehensive Cancer Center (RPCCC). Our study incorporated cardiac tissue histology, quantification of fibrosis and cardiomyocyte nuclei numbers, and second harmonic generation (SHG) imaging to analyze collagen fiber orientation. Additionally, we examined cardiac function, including ejection fraction, and biomarkers such as B-type natriuretic peptide, cardiac troponin, and creatine-kinase MB. Further analyses included additional aspects of myocardial morphology as well as demographics. By incorporating histological findings, cardiac biomarkers and imaging data, our research aims to offer novel insights into the cardiac effects of HMs.

## Methods

### Study design and data abstraction

This study’s cohort comprises a total of 362 individuals with a history of cancer. Among them, 198 patients have a history of hematological malignancies, and 164 have a history of solid tumors. All the data, including post-mortem reports for these individuals, were obtained from patient records at Roswell Park Comprehensive Cancer Center.

For the cancer statistical information, pre-extracted data was received from the Biomedical Research Informatics Shared Resource at RPCCC. This dataset includes basic demographic information such as race, gender, and tobacco usage history, as well as detailed information about cancer types and cancer treatment history.

This data was further supplemented with additional demographic information, cardiac functional data such as ejection fraction, and cardiac biomarker data, spanning B-type natriuretic peptide, troponin I, and creatine kinase-MB extracted from the RPCCC Electronic Health Record and Cerner databases. It also included cell biomarkers obtained from flow cytometry and immunohistochemical staining collected during the time patients were treated at RPCCC.

The cohort of solid tumors included in this study was composed of patients for whom consent was obtained for an autopsy at RPCCC. Consequently, the distribution of solid tumor types reflects the specific patient population that underwent autopsy at this center rather than the general population.

### Tissue feature extraction methodology

Extraction of tissue features was performed strictly adhering to guidelines set forth by an expert pathologist. Each feature was examined individually, with a grading scheme being determined only after a broad examination of the tissue. Tissue features were extracted by assessing whole slide images of heart tissue stained with H&E. Unless otherwise specified, when available, each whole slide image had at least 4 regions of 1 mm^2^ or greater area were examined before determining a final grade. Grading scales are available in the Appendix 1 for the 4 categorical tissue features we assessed: cytoplasmic vacuoles, nuclear atypia, lipofuscin deposition, and myocyte apposition. Additional considerations for nuclear atypia are discussed in the following paragraph.

### Nuclear atypia

Regions of whole slide images were selected for the quantification of atypical nuclei. Each region’s area was recorded, and the number of atypical nuclei counted. Using the recorded area, the number of atypical nuclei per mm^2^ was calculated. The number of atypical nuclei was then graded on a scale: 0 per mm^2^ = 0, 1–2 per mm^2^ = 1, 3 + per mm^2^ = 2.

### Cardiomyocyte nuclei count

1000 × 1000 pixel sections of H&E-stained whole slide images were selected for regions that contained longitudinal sections when available. Each section was digitally split into a 4 × 4 grid using Irfanview (v4.60 – 64 bit) with the cardiomyocyte nuclei being counted in the top-left square first. Counting then moved horizontally right to the end of the row before proceeding to the next row. The count for each square was recorded, with the total for the full 4 × 4 grid being counted as the final number of cardiomyocyte nuclei for that section. To avoid double-counting, when nuclei were on the edges of a square, they were only counted for that square if they were on the bottom or right edge of the square.

### Cardiomyocyte minor axis length

Formalin-fixed, paraffin-embedded heart Sects. (5 µm) were deparaffinized, rehydrated, and stained with H&E. Staining images were obtained at 20X magnification using an Aperio ScanScope XT and analyzed for cardiomyocyte size. Round-shaped cardiomyocytes were identified, and their diameter was measured using the “ruler tool-F4” in Aperio ImageScope. 50–100 cells were counted per slide, selecting only round, regular-shaped cardiomyocytes present on the section.

### Interstitial fibrosis

Visualization of interstitial fibrosis was achieved through trichrome staining with an Epredia™ Richard-Allan Scientific™ Masson Trichrome Kit and performed by both at our lab and RPCCC’s shared resources using our lab’s protocol. Each slide was scanned at 20 × magnification using Aperio ScanScope XT with the assistance of RPCCC’s shared resources who then shared the scanned digital images in the.svs file format. We applied Aperio ImageScope [v12.4.6.5003] to digitally exclude non-interstitial fibrosis such as perivascular fibrosis. Using the built-in Positive Pixel Count v9 algorithm, the annotated regions were analyzed to quantify the interstitial fibrosis, receiving a number that was then subtracted from 1 to calculate a decimal number representing the percent fibrosis in each analyzed region, a methodology we have previously reported in more detail [11].

### Collagen fiber alignment

#### Image acquisition

The trichrome-stained slides were annotated by an expert pathologist who identified regions of interest for each slide. These regions were imaged with a Leica SP8 Multiphoton microscope. Annotated regions were located and initially imaged with fluorescence imaging at 20 × magnification to establish optimal focus levels. Subsequently, the same regions were imaged with SHG imaging. This type of imaging is especially well-suited for collagen’s non-centrosymmetric structure [12]. This tissue property helps highlight collagen as opposed to other tissue structures.

#### Data preprocessing

Once the images were acquired and the tiles merged, files were saved as.lif files. The.lif files were subsequently opened with ImageJ (1.54f), extracting the SHG images as.tif files. The.tif files were then opened in IrfanView (v4.60 – 64 bit) and had the “Auto-adjust colors” feature applied, improving visual contrast. The resulting images were processed with GIMP (v2.10.36) to remove or blacken collagen areas not associated with interstitial fibrosis such as perivascular fibrosis.

#### CurveAlign analytical methods

The next step was to quantify the alignment of the collagen fibers. CurveAlign (v5.0 Beta) is a program that leverages curvelet transforms to calculate the alignment of collagen fibers in SHG images [13]. Angles are based on an absolute system where 0 degrees is to the right, 90 degrees is up, and 180 degrees is left, much like a unit circle. Individual fiber alignments are determined, and the overall alignment is determined by a mean resultant vector length. The range of the scores provided by CurveAlign is 0 to 1, with 1 being perfect alignment and 0 being no alignment.

#### CurveAlign settings and data reporting

We used the recommended setting of 0.006 for the fraction of coefficients and adjusted the curvelet group radius to 18. The scale was adjusted on a per-image basis to best align the fibers visually to the images. Images were then cropped to representative 1000 × 1000 pixel areas, a little under the recommended upper limit of area size of 1024 × 1024 for CurveAlign. If the images contained visible heterogeneity that could not be captured through isolating a single 1000 × 1000 area, two areas were quantified, and the results averaged to ensure a more robust representation. Alignments were recorded in their original 2-decimal format (e.g., 0.42).

### Antineoplastic therapy classes

To aid in analysis, antineoplastic therapies were split into 7 classes as summarized in the Appendix 1. Each class represents a grouping of therapies with similar mechanisms of action except for Class 7, which encompasses the remaining ungrouped therapies that did not fit into the first 6 classes. A full list of the constituents of each antineoplastic therapy class used in this study is available in the Appendix 1.

### Statistical analyses

Statistical analysis was performed using Welch Two Sample t-test, Fisher's Exact Test for count data with simulated *p*-value (based on 2000 replicates), Pearson's Chi-squared test, and Wilcoxon rank sum exact test. Findings were reported as mean (standard deviation) or n (%) as appropriate. Analysis of collagen fiber alignment and interstitial fibrosis excluded patients with a history of myocardial infarction to remove it as a confounding factor. Cox Proportional Hazards (CPH) was performed with the lifelines package in Python [14]. The time duration was defined as survival time after cancer diagnosis, and the event of interest was cardiac death. This analysis was conducted for all cancer patients, as well as for a subset that included only patients with hematological malignancies. To handle missing data within the dataset, median imputation was applied to all continuous variables using the SimpleImputer from the scikit-learn library. This approach ensured that all observations, regardless of missing values, could be included in the CPH analyses. Initially, a univariate analysis was conducted to identify statistically relevant variables. Variables were excluded if their hazard ratios exceeded 20 or if their confidence intervals ranged beyond 2000, ensuring that only stable and interpretable variables were reported and used for subsequent analysis. Additionally, the set of variables identified as significant in each univariate analysis was refined to enhance model validity. This involved removing correlated variables, excluding those with low sample sizes, and refining the set by focusing on more relevant variables. This approach also helped to align the number of events with the number of variables being examined. Multivariate analysis was then performed using the remaining variables to produce the final results. Findings were recorded as hazard ratios, confidence intervals, p-values for the variables, and the concordance index, along with p-values for the CPH models.

## Results

A total of 362 patients (198 hematological malignancy, 164 solid tumors) individuals with a history of cancer and cancer treatment from September 2000 to September 2021 at Roswell Park Comprehensive Cancer Center were included this study.

### Demographic characteristics

Demographic parameters are summarized in Table 1. The overall average age of patients was 60. Of the 362 patients, 134 were female and 228 were male. Additionally, the most common type of cancer was AML with 93 patients (a full report of the cancer types is available in Appendix 1: Tables 6 and 7).

**Table 1:** Comparison: hematological malignancies (HMs) vs solid tumors (STs)

	**HM** **(*n*** **= 198)**^**a**^	**ST** **(*n*** **= 164)**^**a**^	***p*** **Value**
Demographic parameters			
Age (yrs)	58 (14)	62 (13)	0.007
Race			0.2
White	161 (81%)	120 (73%)	
Black	21 (11%)	20 (12%)	
Hispanic	4 (2.0%)	4 (2.4%)	
Other Race	12 (6.1%)	20 (12%)	
Female	70 (35%)	64 (39%)	0.5
Body mass index (kg/m2)	30 (9)	28 (8)	0.022
Tobacco use	115 (62%)	115 (74%)	0.023
Cardiac biomarkers			
Ejection fraction (%)	59 (15)	64 (12)	0.090
B-type natriuretic peptide (pg/ml)	2,275 (4,279)	867 (1,317)	< 0.001
Troponin I (ng/ml)	1.7 (4.8)	4.6 (17.2)	0.13
Creatine Kinase MB (ng/ml)	7 (17)	12 (29)	0.2
Hemoglobin (g/dl)	6.71 (1.36)	8.06 (1.97)	< 0.001
Post-mortem parameters			
Atherosclerosis (more than mild)	72 (43%)	74 (50%)	0.2
Heart weight (g)	487 (126)	440 (129)	0.001
Left ventricle thickness (cm)	1.62 (0.36)	1.63 (0.41)	0.9
Right ventricle thickness (cm)	0.55 (0.21)	0.53 (0.23)	0.4
Septal wall thickness (cm)	1.51 (0.45)	1.50 (0.39)	0.8
Myocardial morphology			
Collagen fiber alignment	0.71 (0.23)	0.83 (0.12)	0.027
Interstitial fibrosis (%)	0.25 (0.10)	0.26 (0.12)	0.9
Cardiomyocyte nuclei (#)	56 (19)	72 (23)	0.002
Cardiomyocyte minor axis length (µm)	25 (3)	25 (3)	0.42

^a^Values are mean (SD); *n* (%)

**Table 2:** Frequency of cardiac death causes in patients with hematological malignancies (HMs) and solid tumors (STs)

**Cause of Death**	**HM**	**ST**	**Total**
Cardiac arrest	8	10	18
Cardiopulmonary arrest	5	13	18
Cardiac failure	2	1	3
Cardiogenic shock	2	1	3
Atrial fibrillation	1	1	2
Cardiac arrythmia	0	1	1
Cardiac dysrhythmia	1	0	1
Cardiac shock	1	0	1
Unspecified cardiac event	1	0	1
Total	21	27	48

Comparing patients with HMs vs patients with STs, the average age was lower for patients with HMs (58 vs 62, *p* = 0.007) while BMI was higher for patients with HMs (30 kg/m^2^ vs 28 kg/m^2^, *p* = 0.022). While race was not found to be significantly different between the groups, patients with HMs were less likely to have used tobacco products (62% vs 74%, *p* = 0.023).

### Cardiac function, heart, and blood biomarkers

Data indicated no significant difference in ejection fraction, but it trended lower in HM patients than in ST patients (62% vs 67%, *p* = 0.09). Examination of heart and blood biomarkers yielded a higher level of B-type natriuretic peptide for HM patients than ST patients (2,275 pg/ml vs 867 pg/ml, *p* < 0.001) while troponin I and creatine kinase MB levels were not significantly different. Investigation of hemoglobin levels found lower levels for HM patients than for ST patients (6.71 g/dl vs 8.06 g/dl, *p* < 0.001). These data are reported in Table 1.

### Overview of causes of death in the cohort

In our analysis of patient outcomes, the causes of death, when noted, included various complications related to malignancies, respiratory failure, acute respiratory distress syndrome, septic shock, and multiple organ failure due to metastatic cancers. Notably, cardiac complications also contributed to mortality in this patient population. The specific distribution and types of cardiac deaths are detailed in Table 2.

### Cardiac mortality predictors in hematological malignancies

Univariate Cox Proportional Hazards analysis for cardiac death in patients with HMs and STs indicated that BMI, diastolic blood pressure, troponin I levels, presence of atherosclerosis greater than mild, heart weight, right ventricle thickness, treatment with anthracyclines and topoisomerase inhibitors, targeted therapy antineoplastics, radiotherapy, and terminal deoxynucleotidyl transferase (TdT) expression were all significant factors in the risk of cardiac death. When multivariate analysis was performed, high BMI (*p* = 0.02), troponin I levels (*p* = 0.002), and right ventricle thickness (*p* = 0.009) were associated with an increased risk of cardiac death. After reducing the variables to improve model validity, multivariate analysis showed higher BMI (*p *< 0.001), troponin I levels (*p *= 0.002), and right ventricle thickness (*p *= 0.013) were associated with an increased risk of cardiac death. In contrast, the absence of anthracyclines and topoisomerase inhibitors (*p *= 0.041) and lower diastolic blood pressure (*p *= 0.030) were associated with increased risk. These data were reported with a concordance index of 0.74 and a statistical significance of *p* < 0.0001 (Table 3).

**Table 3:** Cox Proportional Hazards for cardiac death among patients with cancer. Confidence: *p* < 0.0001, Concordance index: 0.74 for the Multivariate analysis

	**Univariate**			**Multivariate**		
	**HR**	**CI**	***p*** **Value**	**HR**	**CI**	***p*** **Value**
Demographics						
Age (*n* = 111)	1.00	0.98 – 1.03	0.88			
Black vs White (*n* = 111)	1.14	0.48 – 2.71	0.77			
Female (*n* = 111)	1.07	0.60 – 1.89	0.82			
BMI (*n* = 93)	1.04	1.01 – 1.07	0.003	1.06	1.03 – 1.1	< 0.001
Tobacco use (*n* = 104)	1.36	0.74 – 2.51	0.32			
Cardiac biomarkers						
Ejection fraction (*n* = 71)	0.99	0.96 – 1.01	0.21			
Systolic blood pressure (*n* = 62)	0.99	0.97 – 1.02	0.51			
Diastolic blood pressure (*n* = 62)	0.97	0.94 – 1.0	0.022	0.97	0.94 – 1.0	0.030
B-type natriuretic peptide (*n* = 82)	1.00	1.0 – 1.0	0.73			
Troponin I (*n* = 83)	1.05	1.02 – 1.09	0.002	1.07	1.02 – 1.11	0.002
Creatine-kinase MB (*n* = 65)	1.00	1.0 – 1.02	0.067			
Hemoglobin (*n* = 94)	1.14	0.96 – 1.35	0.14			
Post-mortem parameters						
Atherosclerosis (*n* = 101)	2.05	1.15 – 3.63	0.014			
Heart weight (*n* = 97)	1.00	1.0 – 1.01	0.018			
Left ventricle thickness (*n* = 98)	2.35	0.98 – 5.64	0.056			
Right ventricle thickness (*n* = 98)	4.09	1.53 – 10.99	0.005	3.68	1.32 – 10.29	0.013
Septal wall thickness (*n* = 49)	1.07	0.29 – 3.94	0.92			
Left ventricle mass (*n* = 33)	0.99	0.99 – 1.01	0.53			
Zonal fibrosis (*n* = 50)	1.1	0.77 – 1.57	0.60			
Myocardial morphology						
Interstitial fibrosis (*n* = 42)	3.00	0.01 – 1164.84	0.72			
Cardiomyocyte nuclei number (*n* = 45)	0.99	0.96 – 1.02	0.59			
Cardiomyocyte minor axis length (*n* = 41)	0.85	0.7 – 1.01	0.071			
Nuclear atypia (*n* = 50)	1.78	0.89 – 3.55	0.10			
Lipofuscin deposition (*n* = 50)	0.43	0.18 – 1.02	0.055			
Myocyte apposition (*n* = 50)	0.87	0.29 – 2.61	0.80			
Cancer therapy						
Chemotherapy (*n* = 63)						
Class 1	0.71	0.37 – 1.36	0.30			
Class 2	0.89	0.45 – 1.78	0.75			
Class 3	0.92	0.5 – 1.7	0.79			
Class 4	0.44	0.2 – 1.93	0.033	0.37	0.14 – 0.96	0.041
Class 5	0.41	0.18 – 0.98	0.045			
Class 6	0.48	0.19 – 1.21	0.12			
Class 7	0.75	0.33 – 1.68	0.48			
Radiotherapy (*n* = 111)	0.33	0.17 – 0.64	0.001			
Cell biomarkers						
BCL1 (*n* = 4)	0.24	0.03 – 1.76	0.16			
CD3 (*n* = 29)	2.93	0.4 – 21.32	0.29			
CD5 (*n* = 8)	0.23	0.04 – 1.52	0.13			
CD10 (*n* = 6)	0.42	0.06 – 2.87	0.38			
CD21 (*n* = 2)	3.63	0.06 – 217.06	0.54			
CD23 (*n* = 7)	1.22	0.29 – 5.04	0.79			
CD30 (*n* = 6)	0.38	0.06 – 2.4	0.31			
C-MYC (*n* = 5)	0.36	0.09 – 1.52	0.16			
MUM1 (*n* = 7)	0.55	0.13 – 2.27	0.41			
Blast % (*n* = 17)	0.99	0.94 – 1.04	0.69			
Lysozyme (*n* = 12)	0.55	0.13 – 2.32	0.41			
MPO/MPX (*n* = 23)	0.56	0.08 – 4.09	0.57			
TdT (*n* = 12)	0.23	0.07 – 0.76	0.016			
PAX5 (*n* = 23)	0.48	0.19 – 1.24	0.13			

The analysis includes a total of 111 patients with known causes of death, of which 48 experienced a cardiac death event

Focusing only on patients with HMs, the univariate analysis indicated that diastolic blood pressure, troponin I levels, heart weight, right ventricular thickness, left ventricular mass, treatment with alkylating agents, treatment with anthracyclines and topoisomerase inhibitors, TdT expression, and PAX5 expression were all significant factors in the risk of cardiac death. Following a reduction of variables to enhance model validity, the multivariate analysis revealed higher troponin I levels (*p* < 0.001), greater right ventricular thickness (*p* < 0.001), and lower diastolic blood pressure (*p* < 0.001) were associated with an increased risk of cardiac death. These data were reported with a concordance index of 0.83 and a statistical significance of *p* < 0.0001 (Table 4).

**Table 4:** Cox Proportional Hazards for cardiac death among patients with hematological malignancies. Confidence: *p* < 0.0001, Concordance index: 0.83 for the Multivariate analysis

	**Univariate**			**Multivariate**		
	**HR**	**CI**	***p*** **Value**	**HR**	**CI**	***p*** **Value**
Demographics						
Age (*n* = 52)	1.04	0.99 – 1.09	0.10			
Female (*n* = 52)	1.13	0.45 – 2.83	0.80			
BMI (*n* = 46)	1.03	1.0 – 1.07	0.057			
Tobacco use (*n* = 49)	1.21	0.49 – 2.98	0.67			
Cardiac biomarkers						
Ejection fraction (*n* = 37)	0.98	0.95 – 1.0	0.11			
Systolic blood pressure (*n* = 34)	0.98	0.94 – 1.02	0.24			
Diastolic blood pressure (*n* = 34)	0.95	0.91 – 1.0	0.032	0.90	0.86 – 0.95	< 0.001
B-type natriuretic peptide (*n* = 44)	1.00	1.0 – 1.0	0.85			
Troponin I (*n* = 42)	1.05	1.01 – 1.1	0.016	1.14	1.07 – 1.22	< 0.001
Creatine-kinase MB (*n* = 38)	1.01	1.0 – 1.02	0.084			
Hemoglobin (*n* = 46)	1.27	0.79 – 2.03	0.32			
Post-mortem parameters						
Atherosclerosis (*n* = 47)	2.36	0.9 – 6.17	0.080			
Heart weight (*n* = 46)	1.00	1.0 – 1.01	0.037			
Left ventricle thickness (*n* = 47)	1.86	0.36 – 9.56	0.46			
Right ventricle thickness (*n* = 47)	9.34	2.07 – 42.24	0.004	18.95	3.64 – 98.75	< 0.001
Septal wall thickness (*n* = 24)	4.26	0.39 – 46.82	0.24			
Left ventricle mass (*n* = 16)	1.01	1.01 – 1.02	0.021			
Zonal fibrosis (*n* = 34)	1.07	0.67 – 1.7	0.78			
Myocardial morphology						
Interstitial fibrosis (*n* = 27)	0.05	0.0 – 161.34	0.47			
Cardiomyocyte nuclei number (*n* = 29)	0.99	0.95 – 1.03	0.53			
Cardiomyocyte minor axis length (*n* = 14)	0.69	0.44 – 1.08	0.10			
Nuclear atypia (*n* = 34)	1.76	0.76 – 4.08	0.19			
Lipofuscin deposition (*n* = 34)	1.12	0.4 – 3.15	0.83			
Myocyte apposition (*n* = 34)	0.62	0.12 – 3.3	0.58			
Cancer therapy						
Chemotherapy (*n* = 42)						
Class 1	0.34	0.14 – 0.83	0.018			
Class 2	1.1	0.4 – 3.01	0.85			
Class 3	0.58	0.23 – 1.47	0.25			
Class 4	0.35	0.13 – 0.98	0.045			
Class 5	0.47	0.15 – 1.43	0.81			
Class 6	0.31	0.04 – 2.38	0.26			
Class 7	1.23	0.48 – 3.19	0.67			
Radiotherapy (*n* = 52)	0.58	0.22 – 1.56	0.28			
Cell biomarkers						
BCL1 (*n* = 4)	0.16	0.02 – 1.34	0.092			
CD3 (*n* = 29)	2.52	0.4 – 21.32	0.29			
CD5 (*n* = 8)	0.21	0.03 – 1.55	0.12			
CD10 (*n* = 6)	0.35	0.05 – 2.71	0.31			
CD21 (*n* = 2)	4.82	0.06 – 419.71	0.49			
CD23 (*n* = 7)	1.42	0.32 – 6.25	0.64			
CD30 (*n* = 6)	0.24	0.03 – 1.74	0.16			
C-MYC (*n* = 5)	0.27	0.06 – 1.23	0.091			
MUM1 (*n* = 7)	0.48	0.11 – 2.11	0.33			
Blast % (*n* = 17)	0.99	0.95 – 1.04	0.83			
Lysozyme (*n* = 12)	0.5	0.11 – 2.33	0.38			
MPO/MPX (*n* = 23)	0.43	0.06 – 3.31	0.42			
TdT (*n* = 12)	0.13	0.03 – 0.51	0.003			
PAX5 (*n* = 23)	0.34	0.12 – 0.96	0.043			

This analysis includes 52 patients with known causes of death, 21 of whom experienced cardiac death

### Post-mortem analysis of cardiac morphology

**Fig. 1:**
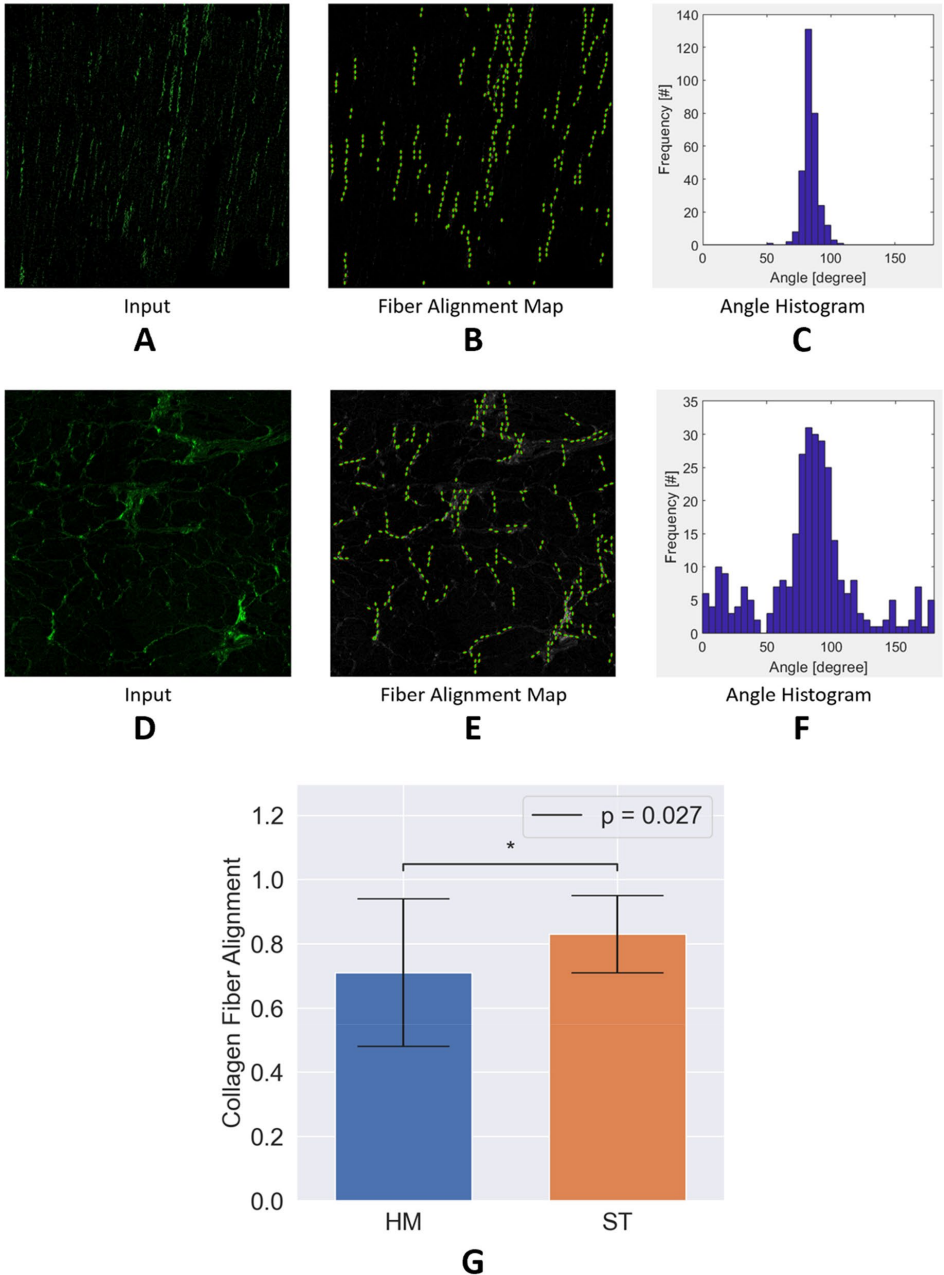
Representative images showing quantification of collagen fiber alignment in second harmonic generation (SHG) images of myocardial tissue sections using CurveAlign. The upper panels show representative images from well-aligned collagen fibers in the myocardium. The tissue fibers were quantified by CurveAlign on a scale of 0–1 where 1 is perfect alignment and lower numbers indicate less alignment. **A** Image with an alignment of 0.98, indicating near-perfect alignment. **B** The Fiber Alignment Map shows the individual fiber alignments which are captured in aggregate and visualized in the Angle Histogram (**C**). The very concentrated nature of the angle distribution indicates strong agreement with the high alignment quantified by CurveAlign. The lower panels show representative images from poorly aligned collagen fibers in the myocardium. **D** Image with an alignment of 0.48, indicating poor alignment. **E** The Fiber Alignment Map shows the individual fiber alignments which are captured in aggregate and visualized in the Angle Histogram. **F** The wide variety of angles seen in the Angle Histogram agrees with the lower score assigned by CurveAlign. **G** Collagen fiber alignment for the Hematological Malignancy (HM) group is compared to the Solid Tumor (ST) group, showing significantly lower alignment in the HM group as indicated by the 0.027 *p*-value. The Fiber Alignment Map and Angle Histogram were created as output images by CurveAlign [13]. The Inputs (Panels **A** & **D**) were slightly brightened for visualization purposes. Magnification 20X

Post-mortem analysis is reported in Table 1. Collagen fiber alignment was found to be lower in the HM group as opposed to the ST group (0.71 vs 0.83, *p* = 0.027), Fig. 1. Additionally, quantification of cardiomyocyte nuclei showed that the HM group had lower counts than the ST group (56 vs 72, *p* = 0.002). Interstitial fibrosis and cardiomyocyte minor axis length were also examined but did not yield a significant difference between the groups.

Examination of post-mortem cardiac specimens yielded heavier heart weight for patients with HMs vs STs (487 g vs 440 g, *p* = 0.001). However, there was no difference in the thickness of the left ventricle, right ventricle, and septal wall. Additionally, occurrence of atherosclerosis more severe than mild was not significantly different.

Additional tissue features were also investigated but out of cytoplasmic vacuoles, nuclei atypia, lipofuscin deposition, and myocyte apposition, only myocyte apposition yielded a significant difference with the ST group having more apposition than the HM group (p = 0.011). This data is reported in Appendix 1: Table 8.

### *Cancer* therapy analyses

The majority of patients (*n* = 250) received systemic cancer therapy without surgery. A subset of solid tumor patients received neoadjuvant therapy prior to surgery (*n* = 7) and another subset received adjuvant therapy after surgery (*n* = 18). Additionally, a few patients (*n* = 6) received both adjuvant therapy and systemic therapy without surgery in either order. One patient received systemic therapy both before and after surgery.

Patients with HM were more likely than ST patients to be treated with cytotoxic agents (77% vs 2.0%, *p* < 0.001), anthracyclines or other topoisomerase inhibitors (55% vs 38%, *p* = 0.040), and therapies outside of our six major therapy classes (39% vs 18%, *p* = 0.007). On the contrary, ST patients were more likely than HM patients to be treated with anti-mitotic agents (30% vs 22%, *p* = 0.040) and radiotherapy (47% vs 30%, *p* = 0.007). The other groups of therapies were not significantly different in use between the groups. These data are reported in Appendix 1: Table 9.

### HM subtype analysis

Majority of HM patients in this cohort had AML. Therefore, subtype analyses were focused on patients with AML. Analysis showed that AML patients were younger than ST patients (58 vs 62, *p* = 0.032), mirroring the trend of the full HM group. Similarly, AML patients had higher BMI than ST patients (31 kg/m^2^ vs 28 kg/m^2^, *p* = 0.027). However, no significant differences were found in race, gender, and tobacco use.

No significant difference was found for ejection fraction, however, BNP (2,422 pg/ml vs 867 pg/ml, *p* = 0.010) was higher for the AML group than the ST group despite troponin I and creatine-kinase MB not being significantly different. Hemoglobin levels were also lower for the AML group than the ST group (6.73 g/dl vs 8.06 g/dl, *p* < 0.001). These results are displayed in Table 5.

**Table 5:** Comparison: acute myeloid leukemia (AML) vs solid tumors (STs)

	**AML** **(*n* = 93)**^**a**^	**ST** **(*n* = 164)**^**a**^	***p*** **Value**
Demographic parameters			
Age (yrs)	58 (14)	62 (13)	0.032
Race			0.60
White	74 (80%)	120 (73%)	
Black	10 (11%)	20 (12%)	
Hispanic	2 (2.2%)	4 (2.4%)	
Other Race	7 (7.5%)	20 (12%)	
Female	29 (31%)	64 (39%)	0.20
Body mass index (kg/m2)	31 (9)	28 (8)	0.027
Tobacco use	55 (63%)	115 (74%)	0.067
Cardiac biomarkers			
Ejection fraction (%)	62 (11)	64 (12)	0.60
B-type natriuretic peptide (pg/ml)	2,422 (4,770)	867 (1,317)	0.010
Troponin I (ng/ml)	0.9 (2.4)	4.6 (17.2)	0.055
Creatine Kinase MB (ng/ml)	5 (11)	12 (29)	0.067
Hemoglobin (g/dl)	6.73 (0.95)	8.06 (1.97)	< 0.001
Post-mortem parameters			
Atherosclerosis (more than mild)	31 (40%)	74 (50%)	0.20
Heart weight (g)	494 (110)	440 (129)	0.001
Left ventricle thickness (cm)	1.71 (0.39)	1.63 (0.41)	0.19
Right ventricle thickness (cm)	0.60 (0.25)	0.52 (0.23)	0.039
Septal wall thickness (cm)	1.52 (0.37)	1.50 (0.39)	0.82
Myocardial morphology			
Collagen fiber alignment	0.66 (0.33)	0.83 (0.12)	0.14
Interstitial fibrosis (%)	0.23 (0.09)	0.22 (0.08)	0.97
Cardiomyocyte nuclei (#)	52 (21)	72 (23)	0.002
Cardiomyocyte minor axis length (µm)	27 (4)	25 (3)	0.065

^a^Values are mean (SD); *n* (%)

Comparing myocardial morphology showed that collagen fiber alignment, interstitial fibrosis, and cardiomyocyte minor axis length were not significantly different between patients with AML and patients with STs. However, cardiomyocyte nuclei numbers were lower for the AML patients than the ST patients (52 vs 72, *p* = 0.002). In contrast, no significant differences were found for the other tissue features (Table 5 and Appendix 1: Table 10).

Examination of the post-mortem parameters yielded higher heart weight for AML patients than ST patients (494 g vs 440 g, *p* = 0.001) and thicker right ventricles (0.60 cm vs 0.53 cm, *p* = 0.039). However, left ventricle thickness, septal wall thickness, and instance of atherosclerosis more severe than mild were not significantly different.

### Analysis of the largest homogeneous subtypes between HM and ST

We were also interested in comparing the two largest homogeneous groups among HM and ST: AML and lung cancer (LC), as this would help control for differences in cancer type. These data are displayed in Appendix 1: Table 11.

The LC group had patients who were older than the AML group (58 vs 64, *p* = 0.026) and BMI was lower for the LC group (31 kg/m^2^ vs 26 kg/m^2^, *p* = 0.008). While hemoglobin levels were lower for the AML group than the LC group (7.80 g/dl vs 6.73 g/dl, *p* = 0.041), ejection fraction, BNP levels, troponin I levels, and creatine-kinase MB were not found to be significantly different.

Collagen fiber alignment, interstitial fibrosis, and cardiomyocyte minor axis length did not show any significant differences between the AML and LC groups. Cardiomyocyte nuclei density, however, was lower for the AML patients (52 vs 81, *p* = 0.049).

For post-mortem parameters, AML patients had higher heart weight (494 g vs 440 g, *p* = 0.03) and thicker right ventricles (0.60 cm vs 0.48 cm, *p* = 0.007). Left ventricle thickness, septal wall thickness, and instance of atherosclerosis greater than mild were not significantly different between the groups. Overall, the results for the AML and LC comparison were similar to those of the AML vs ST comparison, with the exception of collagen fiber alignment.

## Discussion

The findings of our study provide critical insights into the cardiovascular complications associated with hematological malignancies. We compared cardiac histomorphology and biomarkers among patients with HMs vs STs, AML vs STs, and AML vs LC. Our results show that HM patients exhibited significantly lower collagen fiber alignment, fewer cardiomyocyte nuclei, and higher levels of B-type natriuretic peptide, indicating an increased risk of ACEs. Additionally, there was no significant difference in interstitial collagen volume fraction between these groups. Subtype analysis revealed that AML patients had a reduced cardiomyocyte nuclei count compared to ST patients, along with elevated BNP levels. Further subtype analysis of AML versus LC revealed that AML patients had significantly fewer cardiomyocyte nuclei compared to LC patients, although BNP levels did not show a significant difference between the groups. Multivariate analysis indicated that higher troponin I levels and right ventricular thickness were associated with an increased risk of cardiac death. Conversely, lower diastolic blood pressure correlated with a higher risk of cardiac death. Our results highlight the elevated concern for ACEs in HM patients.

An increasing association between cardiovascular morbidity and mortality has been reported in cancer patients and survivors [15, 16]. In a community-based ARIC study, cancer survivors had a 37% higher risk of cardiovascular disease and a 52% higher risk of heart failure [17]. Similarly, Strongman et al. noted that patients with hematological malignancies and other types of cancer face a higher risk of developing heart-related conditions, such as coronary artery disease, arrhythmia, pericarditis, valvular heart disease, and stroke [18]. Furthermore, HMs have been linked to increased cardiovascular risks including stroke, acute myocardial infarction, and heart failure [19]. while increased episodes of ventricular arrhythmias was observed in patients with pancreatic, lung, and colorectal cancer [20]. However, unlike our study, previous research has not compared HM and ST patients to assess cardiovascular risks based on histopathological features.

Several studies have documented the increased risk of cardiovascular disease in patients with HMs. Armenian et al. observed a heightened incidence of heart failure and myocardial infarction among survivors of AML, attributing this increased risk to both the malignancy itself and the cardiotoxic effects of its treatment [21]. Similarly, Boluda and colleagues reported that patients with HMs, including AML, are predisposed to cardiac events, partly due to intensive chemotherapy and the associated inflammatory response [4]. Our study corroborates these findings by demonstrating that HM patients, particularly those with AML, exhibit significant cardiac morphological changes. We observed a marked reduction in cardiomyocyte nuclear density in HM patients compared to those with STs, indicating potential cardiomyocyte loss or damage. This finding aligns with the research by Johnson et al., which reported similar myocardial damage in patients undergoing treatment for AML [22].

Our study found that HM patients have an increased risk of ACEs compared to those with STs. Support for this hypothesis comes from the presence of myocardial dysfunction in acute leukemia patients compared to STs and non-cancer patients [6]. Similarly, the risk of heart failure was higher among hematopoietic and lymphatic cancers compared to STs [23]. In contrast, a different study involving 5.9 million US patients admitted to hospitals for cardiovascular reasons, revealed that heart failure was most common in patients with hematological malignancies, while acute myocardial infarction was associated with colon cancer, and atrial fibrillation occurred frequently in patients with lung cancer [24]. Another study found that a plurality of cardiovascular disease deaths in patients under the age of 40 were attributable to breast cancer and lymphomas while the deaths were attributable to lung, breast, colorectal cancer and prostate cancer for patients 40 and older [25]. Our data showed that patients with HMs were younger than those with STs supporting the disparity in age-based mortality. Furthermore, another study observed the development of cardiovascular disease, causing excess mortality in long-term survivors of lymphoma [26]. Our findings are consistent with these differences in cardiovascular complications between HMs and STs.

Additionally, our study identified elevated levels of B-type natriuretic peptide, biomarker indicative of cardiac stress, in HM patients. These results are consistent with previous studies that have associated elevated BNP levels with poor cardiac outcomes in cancer patients. Maniu et al., have shown that leukemia patients had values higher than the normal limits for CK-MB and NT-proBNP [27]. Another study found the mean value of troponin I was significantly greater in patients with hematological malignancies [28]. In our study, troponin I trended higher in HM patients, but the results were not statistically significant; however, it was still identified as a significant factor in our analysis of cardiac death risk. Additionally, unlike our study, which found no significant difference in creatine-kinase MB levels, some research has reported increased levels of this enzyme in HM patients after therapy, suggesting variability in myocardial damage markers depending on the patient population and specific treatments used [29].

Similarly, HM patients had significantly lower hemoglobin levels and cardiomyocyte nuclei counts, suggesting more severe cardiac and systemic impact of HMs compared to solid tumors. This finding is partially supported by the work of Ludwig et al., who found that anemia was more common in cancer patients treated with chemotherapy, which could exacerbate cardiac dysfunction in in these patients [30]. Moreover, the higher BNP levels in HM patients further underscore the greater cardiac stress and injury in this group. Multivariate analysis identified low ejection fraction, older age, high BMI and increased LV mass as significant risk factors for cardiac death in HM patients. These findings align with the broader literature on cancer-related cardiac risk factors, which frequently cites reduced cardiac function and obesity as major contributors to cardiac mortality in cancer patients [31]. Depletion of cardiomyocyte nuclei has been associated with treatment with anthracyclines [32]. This could explain the impact on HM patients due to their higher occurrence of treatment with anthracyclines.

Elevated collagen levels, combined with degeneration of cardiomyocytes, result in myocardial fibrosis and stiffness, potentially leading to cardiac dysfunction [33]. Less alignment of collagen fibers has been hypothesized to be associated with worse electromechanical dysfunction [34]. While there was increased collagen fiber disarray in HM patients, there was no difference in myocardial interstitial collagen volume fraction between HM and ST patients.

Mechanistically speaking, hematological malignancies produce an excessive amount of reactive oxygen species due to oncogene activation and increased metabolic activity. This leads to cardiometabolic disease due to oxidative stress and chronic inflammation in these patients. Chemotherapy can exacerbate these effects [35]. The direct connection of leukemia to the bloodstream after proliferation in the bone marrow can lead to subsequent circulation to the heart and infiltration of the cardiac tissue. In contrast, lymphoma typically spreads through lymphatic channels rather than directly infiltrating the heart [36]. This distinction partly explains the increased incidence of ACEs in patients with leukemia including AML.

Leukemia patients are treated more frequently with anthracyclines and other topoisomerase inhibitors compared to ST patients, which could also impart a higher risk of cardiotoxicity in these patients. Previous study reported that the absolute excess risks were generally higher with increasing age and chemotherapy [18]. Considering all-cause mortality, cardiovascular risk factors among cancer patients are heart failure, myocardial infarction, atrial fibrillation, stroke, and hypertension and diabetes to a lesser degree [37]. Other factors associated with increased cardiovascular death include older age at diagnosis, local metastasis, surgery, and chemotherapy [38]. Multivariate analysis conducted in our study indicated that reductions in both ejection fraction along with severe atherosclerosis and higher BMI were risk factors for cardiac death among our patient cohort. Although these results did not offer any new insights, they do reinforce the need for monitoring these factors in cancer patients undergoing treatment. Further research is necessary to fully understand the clinical implications and develop measures to mitigate these risks.

## Limitations

This study has several limitations that should be considered. Firstly, its retrospective nature and reliance on post-mortem evaluations may introduce selection biases and affect data accuracy. The relatively small sample size, particularly within specific cancer subgroups, limits the generalizability of the findings. Treatment variability, including differences in chemotherapeutic agents and radiation therapies, was not fully controlled for, potentially influencing cardiac outcomes and confounding the results.

The lack of longitudinal data on cardiac function before, during, and after cancer treatment prevents establishing causal relationships between cancer, its treatment, and cardiac complications. Histopathological analysis does not capture dynamic cardiac injury and repair processes, and post-mortem changes could affect tissue morphology. Biomarker levels may be influenced by comorbid conditions and acute events near the time of death, potentially confounding the results.

The study also did not comprehensively account for comorbidities such as hypertension and diabetes, which could contribute to cardiac complications. Future studies should address these limitations with larger, prospective cohorts, detailed treatment regimens, comprehensive control for comorbidities, and longitudinal data to better understand the relationship between cancer, its treatment, and cardiac complications.

## Conclusion

In conclusion, our study reveals significant cardiac complications in patients with HMs compared to STs. HM patients showed lower collagen fiber alignment, reduced cardiomyocyte nuclei count, and elevated cardiac biomarkers, indicating higher cardiac stress and dysfunction. These findings highlight the need for specialized cardiac care, enhanced monitoring, and individualized treatment strategies for HM patients. Further research should focus on baseline assessments and longitudinal studies to improve patient care and identify factors contributing to cardiotoxicity.

## Figures and Tables

**Fig. 2:**
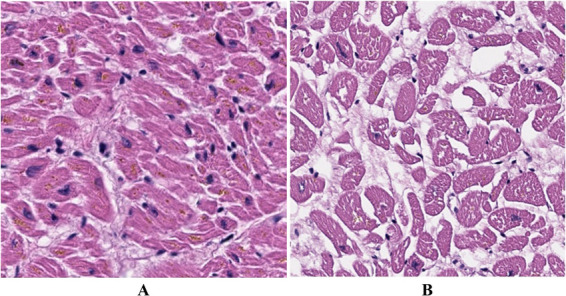
Representative images for nuclear density examined on hematoxylin and eosin-stained sections. **A** High nuclear density in a patient with solid tumor. **B** Low nuclear density in patient with hematological malignancy. Magnification 20X

**Fig. 3:**
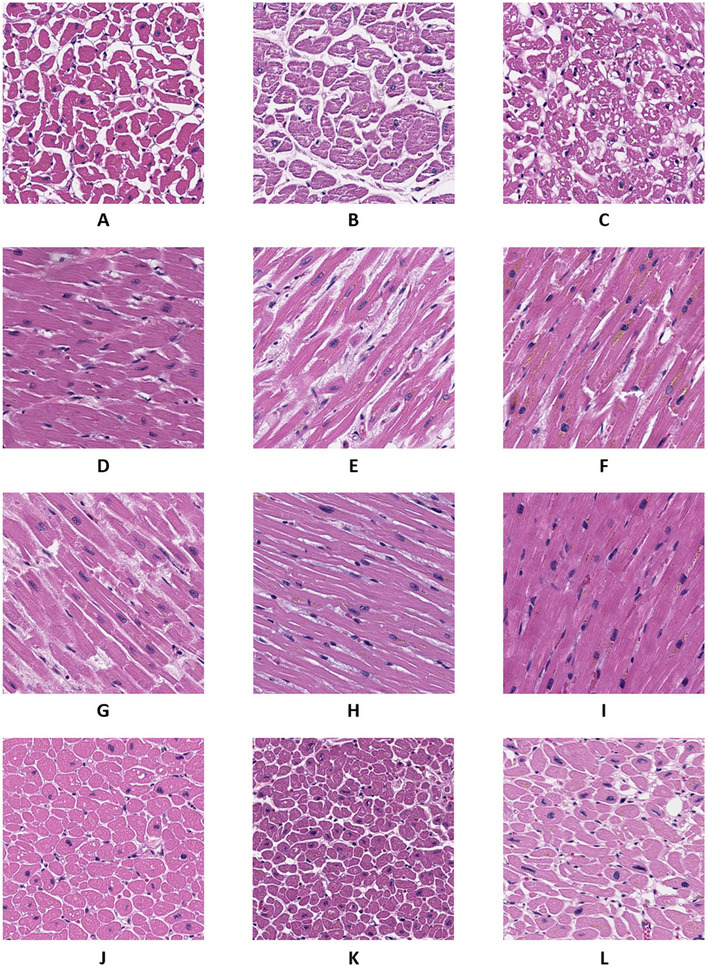
Representative images for cardiac morphological features. Cytoplasmic vacuoles (**A**-**C**), lipofuscin deposition (**D**-**F**), myocyte apposition (**G**-**I**), and nuclear atypia (**J**-**L**) are shown from least severe to most severe, corresponding to the least to the most (grades of 0, 1, and 2) from left and right. Magnification 20X

**Fig. 4:**
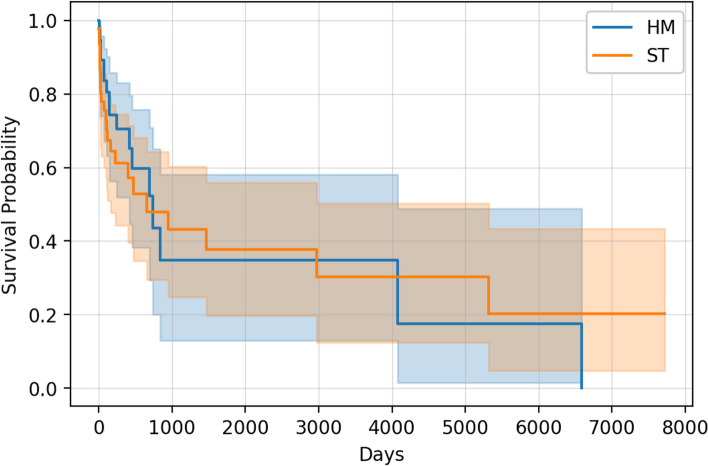
Kaplan-Meier survival curve for hematological malignancies vs solid tumors examining cardiac death in cancer patients. A log-rank test produced *p*=0.74, indicating no significant difference between survival of these two cohorts. Created with lifelines in Python [14]

**Fig. 5:**
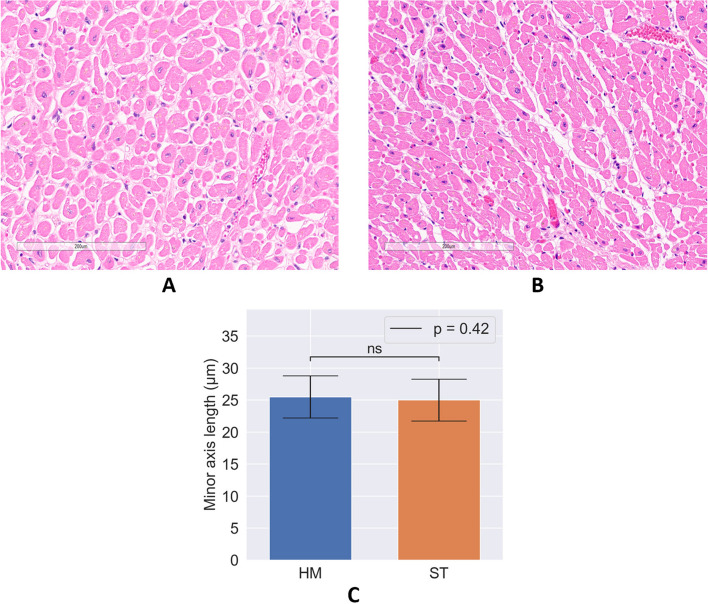
Data showing comparison of cardiomyocyte minor axis length between hematological malignancy (HM) and solid tumor (ST). **A** Representative image for the HM group. **B** Representative image for the ST group. **C** The HM group had an average minor axis length of 25.5 µm while the ST group had an average minor axis length of 25.0 µm. Analysis with a Welch Two Sample t-test indicated a *p*-value of 0.42, indicating no significant difference between the groups

## Data Availability

The data that support the findings of this study are available upon request from the corresponding author.

## Appendix

**Table 6:** Hematological malignancy subtypes

Hematological malignancies (HMs)	**#**
Acute myeloid leukemia	90
Chronic myeloid leukemia	12
Acute lymphoblastic leukemia	14
Chronic lymphocytic leukemia	6
Lymphoma	43
Leukemia, NOS	10
Myeloma	6
Myelodysplastic syndrome	11
Other HMs	1
Multiple HMs	4
One case that was AML or CML but not clear	1
Total HMs	198

**Table 7:** Solid tumor subtypes

Solid tumors (STs)	**#**
Lung cancer	29
Breast cancer	5
Thyroid/papillary thyroid carcinoma	2
Colon/colorectal carcinoma	5
Esophageal carcinoma	13
Ovarian cancer	3
Prostatic carcinoma	5
Urothelial carcinoma	9
Pancreatic carcinoma	5
Cholangiocarcinoma	2
Renal cell carcinoma	3
Hepatocellular carcinoma	6
Adenocarcinoma (unknown primary)	20
Squamous cell carcinoma (unknown primary)	12
Poorly differentiated carcinoma	6
Carcinoma, NOS	4
Sarcoma	7
Melanoma	5
Other STs	19
Multiple STs	4
Total STs	164

**Table 8:** Additional myocardial morphology comparison: hematological malignancies (HMs) vs solid tumors (STs)

	**HM (*****n*****=92)**	**ST (*****n*****=36)**	***p***** Value**
Cytoplasmic Vacuoles			0.058
0 (absent)	83 (90%)	36 (100%)	
1 (mild)	9 (9.8%)	0 (0%)	
2 (moderate/severe)	0 (0%)	0 (0%)	
Nuclear Atypia			0.9
0 (absent)	26 (28%)	10 (29%)	
1 (mild)	54 (59%)	22 (63%)	
2 (moderate/severe)	12 (13%)	3 (8.6%)	
Lipofuscin Deposition			0.5
0 (absent)	1 (1.1%)	0 (0%)	
1 (mild/moderate)	56 (61%)	18 (50%)	
2 (severe)	35 (38%)	18 (50%)	
Myocyte apposition			0.011
0 (absent)	1 (1.1%)	0 (0%)	
1 (mild/moderate)	79 (86%)	24 (67%)	
2 (severe)	12 (13%)	12 (33%)	

The tissue samples that met the acceptable criteria for histologic analysis of specific features were limited, resulting in smaller sample sizes

**Table 9:** Cancer therapy comparison: hematological malignancies (HMs) vs solid tumors (STs)

	**HM (*****n*** **= 144)**	**ST (*****n*****=50)**	***p***** Value**
Cancer Therapy			
Alkylating agents	84 (58%)	30 (60%)	0.8
Cytotoxic agents	111 (77%)	1 (2.0%)	< 0.001
Antimetabolites	70 (49%)	22 (44%)	0.6
Anthracyclines and topoisomerase inhibitors	79 (55%)	19 (38%)	0.040
Targeted therapy	31 (22%)	15 (30%)	0.2
Anti-mitotic agents	24 (17%)	19 (38%)	0.002
Other therapies	56 (39%)	9 (18%)	0.007
Radiotherapy (*n*=198, 164)	59 (30%)	77 (47%)	< 0.001

Alkylating agents: Bendamustine, Busulfan, CARBOplatin, CISplatin, Cyclophosphamide, Dacarbazine, Ifosfamide, Melphalan, Mesna, OXALIplatin, Procarbazine, Temozolomide, Thiotepa, Trabectedin

Cytotoxic agents: Arsenic Trioxide, Bleomycin, Carmustine, Clofarabine, Cytarabine, Decitabine, Fludarabine, IDArubicin, Tretinoin

Antimetabolites: Azacitidine, Cladribine, Fluorouracil, Gemcitabine, Mercaptopurine, Methotrexate, PEMEtrexed, Pentostatin, Thioguanine

Anthracyclines and topoisomerase inhibitors: DAUNOrubicin, DOXOrubicin, Etoposide, Irinotecan, MitoXANTRONE, Topotecan

Targeted therapy: Alemtuzumab, Axitinib, Bevacizumab, Blinatumomab, Brentuximab Vedotin, Cetuximab, Dabrafenib, Dasatinib, Gemtuzumab Ozogamicin, Ibrutinib, Imatinib Mesylate, Necitumumab, Nilotinib, Nivolumab, Pembrolizumab, Pertuzumab, Polatuzumab Vedotin, Ramucirumab, RiTUXimab, Ruxolitinib, Sorafenib, Trastuzumab

Anti-mitotic agents: DOCEtaxel, PACLitaxel, VinBLAStine, vinCRIStine, VinORELbine

Other therapies: Aldesleukin, Asparaginase, Bortezomib, Carfilzomib, Denileukin Diftitox, EriBULin Mesylate, Fulvestrant, Goserelin Acetate, Hydroxyurea, Lenalidomide, Letrozole, Leuprolide, Megestrol Acetate, Pegaspargase, Porfimer, Tamoxifen, Thalidomide, Venetoclax, Zosuquidar

**Table 10:** Additional myocardial morphology comparison: acute myeloid leukemia (AML) vs solid tumors (STs)

	**AML (*****n*****=47)**	**ST (*****n*****=36)**	***p***** Value**
Cytoplasmic Vacuoles			0.067
0 (absent)	42 (89%)	36 (100%)	
1 (mild)	5 (11%)	0 (0%)	
2 (moderate/severe)	0 (0%)	0 (0%)	
Nuclear Atypia			0.4
0 (absent)	11 (23%)	10 (29%)	
1 (mild)	27 (57%)	22 (63%)	
2 (moderate/severe)	9 (19%)	3 (8.6%)	
Lipofuscin Deposition			0.4
0 (absent)	0 (0%)	0 (0%)	
1 (mild/moderate)	29 (62%)	18 (50%)	
2 (severe)	18 (38%)	18 (50%)	
Myocyte apposition			0.13
0 (absent)	0 (0%)	0 (0%)	
1 (mild/moderate)	39 (83%)	24 (67%)	
2 (severe)	8 (17%)	12 (33%)	

The tissue samples that met the acceptable criteria for histologic analysis of specific features were limited, resulting in smaller sample sizes

**Table 11:** Comparison: acute myeloid leukemia (AML) vs lung cancer (LC)

	**AML (*****n*** **= 93)**^**a**^	**LC (*****n*****=29)**^**a**^	***p***** Value**
Demographic parameters			
Age (yrs)	58 (14)	64 (11)	0.026
Race			0.94
White	74 (80%)	22 (76%)	
Black	10 (11%)	4 (14%)	
Hispanic	2 (2.2%)	1 (3.4%)	
Other Race	7 (7.5%)	2 (6.9%)	
Female	29 (31%)	13 (45%)	0.26
Body mass index (kg/m2)	31 (9)	26 (7)	0.008
Tobacco use	55 (62%)	25 (93%)	0.006
Cardiac biomarkers			
Ejection fraction (%)	62 (11)	62 (18)	0.97
B-type natriuretic peptide (pg/ml)	2,422 (4,770)	1,102 (1,790)	0.071
Troponin I (ng/ml)	0.9 (2.4)	5.1 (18.3)	0.37
Creatine Kinase MB (ng/ml)	5 (11)	15 (36)	0.35
Hemoglobin (g/dl)	6.73 (0.95)	7.80 (2.12)	0.041
Post-mortem parameters			
Atherosclerosis (more than mild)	31 (40%)	16 (57%)	0.19
Heart weight (g)	494 (110)	440 (129)	0.030
Left ventricle thickness (cm)	1.71 (0.39)	1.61 (0.49)	0.37
Right ventricle thickness (cm)	0.60 (0.25)	0.48 (0.18)	0.007
Septal wall thickness (cm)	1.52 (0.37)	1.50 (0.39)	0.91
Myocardial morphology			
Collagen fiber alignment	0.66 (0.33)	0.83 (0.02)	0.15
Interstitial fibrosis (%)	0.23 (0.09)	0.20 (0.06)	0.42
Cardiomyocyte nuclei (#)	52 (21)	81 (31)	0.049
Cardiomyocyte minor axis length (µm)	27 (4)	24 (3)	0.093

^a^Values are mean (SD); *n* (%)

**Table 12:** Tissue feature grading. Nuclear atypia was assessed in atypical nuclei per mm^2^

**Tissue Feature**	**Grading**
Cytoplasmic vacuoles	0 absent, 1 mild, 2 moderate/severe.
Nuclear atypia	0 absent, 1 = 1-2, 2 = 3+
Lipofuscin deposition	0 absent, 1 mild/moderate, 2 severe.
Myocyte apposition	0 absent, 1 mild/moderate, 2 severe.

**Table 13:** Classes of antineoplastic therapies

**Classes**	**Mechanism of Action Groupings**
Class 1	Alkylating agents
Class 2	Cytotoxic agents
Class 3	Antimetabolites
Class 4	Anthracyclines and Topoisomerase inhibitors
Class 5	Targeted therapy
Class 6	Anti-mitotic agents
Class 7	Other

## References

[1] Miller KD, et al. Cancer treatment and survivorship statistics, 2022. CA Cancer J Clin. 2022;72(5):409–36. 35736631 10.3322/caac.21731

[2] Kocarnik JM, et al. Cancer Incidence, Mortality, Years of life lost, years lived with disability, and disability-adjusted life years for 29 cancer groups from 2010 to 2019: a systematic analysis for the global burden of disease study 2019. JAMA Oncol. 2022;8(3):420–44. 34967848 10.1001/jamaoncol.2021.6987 PMC8719276

[3] Voigt P, et al. Cardiac hematological malignancies: typical growth patterns, imaging features, and clinical outcome. Angiology. 2018;69(2):170–6. 28602141 10.1177/0003319717713581

[4] Boluda B, et al. Incidence and risk factors for development of cardiac toxicity in adult patients with newly diagnosed acute myeloid leukemia. Cancers (Basel). 2023;15(8);2267. 37190195 10.3390/cancers15082267 PMC10136564

[5] Raisi-Estabragh Z, et al. Temporal trends in disease-specific causes of cardiovascular mortality amongst patients with cancer in the USA between 1999 and 2019. Eur Heart J Qual Care Clin Outcomes. 2022;9(1):54–63. 35435219 10.1093/ehjqcco/qcac016 PMC9745666

[6] Assuncao B, et al. Acute Leukemia is Associated with Cardiac Alterations before Chemotherapy. J Am Soc Echocardiogr. 2017;30(11):1111–8. 28927558 10.1016/j.echo.2017.07.016

[7] Fernandez Botana I, et al. Interleukin-27 tackles immunosuppression in chronic lymphocytic leukemia. Oncoimmunology. 2023;12(1):2276490. 37937211 10.1080/2162402X.2023.2276490 PMC10627055

[8] Zhao Y, et al. Clinical features of cardiac lymphoma: an analysis of 37 cases. J Int Med Res. 2021;49(3):300060521999558. 33752450 10.1177/0300060521999558 PMC7995496

[9] Yasmeen T, et al. Frequency and causes of anemia in Lymphoma patients. Pak J Med Sci. 2019;35(1):61–5. 30881397 10.12669/pjms.35.1.91 PMC6408630

[10] Sanchez-Correa B, et al. Cytokine profiles in acute myeloid leukemia patients at diagnosis: Survival is inversely correlated with IL-6 and directly correlated with IL-10 levels. Cytokine (Philadelphia, Pa). 2013;61(3):885–91. 10.1016/j.cyto.2012.12.023 23357299

[11] Sonkawade SD, et al. Small Endogeneous Peptide Mitigates Myocardial Remodeling in a Mouse Model of Cardioselective Galectin-3 Overexpression. Circ Heart Fail. 2021;14(9):e008510. 34415177 10.1161/CIRCHEARTFAILURE.121.008510 PMC8458256

[12] Campagnola PJ, et al. Three-dimensional high-resolution second-harmonic generation imaging of endogenous structural proteins in biological tissues. Biophys J. 2002;82(1 Pt 1):493–508. 11751336 10.1016/S0006-3495(02)75414-3 PMC1302489

[13] Bredfeldt JS, et al. Automated quantification of aligned collagen for human breast carcinoma prognosis. J Pathol Inform. 2014;5(1):28. 25250186 10.4103/2153-3539.139707 PMC4168643

[14] Davidson-Pilon C. Lifelines: survival analysis in Python. J Open Source Software. 2024;4(40):1317.

[15] Paterson DI, et al. Incident Cardiovascular Disease Among Adults With Cancer: A Population-Based Cohort Study. JACC CardioOncol. 2022;4(1):85–94. 35492824 10.1016/j.jaccao.2022.01.100 PMC9040097

[16] Sturgeon KM, et al. A population-based study of cardiovascular disease mortality risk in US cancer patients. Eur Heart J. 2019;40(48):3889–97. 31761945 10.1093/eurheartj/ehz766 PMC6925383

[17] Florido R, et al. Cardiovascular Disease Risk Among Cancer Survivors: The Atherosclerosis Risk In Communities (ARIC) Study. J Am Coll Cardiol. 2022;80(1):22–32. 35772913 10.1016/j.jacc.2022.04.042 PMC9638987

[18] Strongman H, et al. Medium and long-term risks of specific cardiovascular diseases in survivors of 20 adult cancers: a population-based cohort study using multiple linked UK electronic health records databases. Lancet. 2019;394(10203):1041–54. 31443926 10.1016/S0140-6736(19)31674-5 PMC6857444

[19] Yong JH, et al. Cardiovascular Risk in Patients with Hematological Malignancies: A Systematic Review and Meta-Analysis. Am J Cardiol. 2024;212:80–102. 38042266 10.1016/j.amjcard.2023.11.039

[20] Anker MS, et al. Ventricular tachycardia, premature ventricular contractions, and mortality in unselected patients with lung, colon, or pancreatic cancer: a prospective study. Eur J Heart Fail. 2021;23(1):145–53. 33222388 10.1002/ejhf.2059

[21] Armenian SH, et al. Prediction of cardiovascular disease among hematopoietic cell transplantation survivors. Blood Adv. 2018;2(14):1756–64. 30037802 10.1182/bloodadvances.2018019117 PMC6058239

[22] Johnson IM, et al. Cardiac events in patients with acute myeloid leukemia treated with venetoclax combined with hypomethylating agents. Blood Adv. 2022;6(17):5227–31. 35358999 10.1182/bloodadvances.2022007333 PMC9631636

[23] Yun JP, et al. Risk of atrial fibrillation according to cancer type: a nationwide population-based study. JACC CardioOncol. 2021;3(2):221–32. 34396327 10.1016/j.jaccao.2021.03.006 PMC8352078

[24] Matetic A, et al., Impact of cancer diagnosis on causes and outcomes of 5.9 million US patients with cardiovascular admissions. Int J Cardiol, 2021;341:76–83. 34333019 10.1016/j.ijcard.2021.07.054

[25] Stoltzfus KC, et al. Fatal heart disease among cancer patients. Nat Commun. 2020;11(1):2011. 32332714 10.1038/s41467-020-15639-5 PMC7181822

[26] Castellino SM, et al. Morbidity and mortality in long-term survivors of Hodgkin lymphoma: a report from the Childhood Cancer Survivor Study. Blood. 2011;117(6):1806–16. 21037086 10.1182/blood-2010-04-278796 PMC3056636

[27] Raluca Maniu D, et al. The role of biomarkers and echocardiography in the evaluation of cardiotoxicity risk in children treated for leukemia. J buon. 2018;23(7):122–31. 30722121

[28] Missov E, et al. Cardiac troponin I in patients with hematologic malignancies. Coron Artery Dis. 1997;8(8–9):537–41. 9431482

[29] Omran MM, et al. Imatinib pharmacokinetics and creatine kinase levels in chronic myeloid leukemia patients: implications for therapeutic response and monitoring. Eur J Clin Pharmacol. 2024;80(7):1061–8. 38536418 10.1007/s00228-024-03675-9 PMC11156749

[30] Ludwig H, et al. The European Cancer Anaemia Survey (ECAS): a large, multinational, prospective survey defining the prevalence, incidence, and treatment of anaemia in cancer patients. Eur J Cancer. 2004;40(15):2293–306. 15454256 10.1016/j.ejca.2004.06.019

[31] Wang Y, et al. Cardio-Oncology: a myriad of relationships between cardiovascular disease and cancer. Front Cardiovasc Med. 2022;9:727487. 35369296 10.3389/fcvm.2022.727487 PMC8968416

[32] Xia P, et al. Doxorubicin induces cardiomyocyte apoptosis and atrophy through cyclin-dependent kinase 2-mediated activation of forkhead box O1. J Biol Chem. 2020;295(13):4265–76. 32075913 10.1074/jbc.RA119.011571 PMC7105316

[33] Thiedemann KU, et al. Connective tissue content and myocardial stiffness in pressure overload hypertrophy. A combined study of morphologic, morphometric, biochemical, and mechanical parameters. Basic Res Cardiol. 1983;78(2):140–55. 6223618 10.1007/BF01906668

[34] Mirsanaye K, et al. Polar organization of collagen in human cardiac tissue revealed with polarimetric second-harmonic generation microscopy. Biomed Opt Express. 2019;10(10):5025–30. 31646027 10.1364/BOE.10.005025 PMC6788612

[35] Wu Q, et al. Apigenin ameliorates doxorubicin-induced renal injury via inhibition of oxidative stress and inflammation. Biomed Pharmacother. 2021;137:111308. 33556877 10.1016/j.biopha.2021.111308

[36] Cheng CL, et al. Intralymphatic spread is a rare finding associated with poor prognosis in diffuse large b-cell lymphoma with extranodal involvements. Am J Surg Pathol. 2018;42(5):616–24. 29505426 10.1097/PAS.0000000000001045

[37] Liu D, et al. Prevalence and prognosis significance of cardiovascular disease in cancer patients: a population-based study. Aging (Albany NY). 2019;11(18):7948–60. 31562288 10.18632/aging.102301 PMC6781987

[38] Wang Z, et al. Higher risk of cardiovascular mortality than cancer mortality among long-term cancer survivors. Front Cardiovasc Med. 2023;10:1014400. 36760569 10.3389/fcvm.2023.1014400 PMC9905625

